# A tailored telephone and email based exercise intervention induced reductions in various measures of body composition in physically inactive adults: A randomized controlled trial

**DOI:** 10.1016/j.pmedr.2018.06.011

**Published:** 2018-06-27

**Authors:** Ingirid Geirsdatter Heald Kjær, Sigmund Alfred Anderssen, Monica Klungland Torstveit

**Affiliations:** a Faculty of Health and Sports Science, The University of Agder, Postboks 422, Kristiansand, Norway; b Department of Sport Medicine, The Norwegian School of Sport Sciences, Postboks 4014, Ullevål stadion, 0806 Oslo, Norway

**Keywords:** IG, Intervention Group, CG, Control Group, BMI, Body Mass Index, WC, Waist Circumference, FP^skf^, Fat Percentage by skinfolds, CRF, cardiorespiratory fitness, Mixed delivery modes, Body composition, Weight change

## Abstract

Obesity prevalence has increased the past decades and has become a serious public health problem. The aim of this six-month assessor-blinded, parallel-group randomized controlled trial was to assess the effect of a tailored telephone and email-based exercise intervention on various measures of body composition in a sample of apparently healthy and physically inactive adults. A total of 111 volunteering adults (40–55 yr) in Southern Norway were randomly assigned to an intervention group (IG; n = 39) or a no-information control group (CG; n = 50), by random allocation numbering. The IG received feedback on their health-related physical fitness, information on guidelines and recommendations for physical activity, a leaflet on national dietary recommendations, prompts and reminders in addition to three tailored exercise programs, one every two months, and fortnightly motivational counselling by email or telephone, alternately. The CG received no follow-up during the intervention period. The main outcome measures: weight, body mass index (BMI), waist circumference (WC) and fat percentage by skinfolds (FP^skf^) were assessed objectively at baseline and posttest. A one-way ANCOVA analysis, adjusted for baseline scores, gender, age, and educational level, revealed a larger reduction on all body compositional measures in the IG compared to the CG (p ≤ 0.043), except for BMI when adjusted for baseline scores. Additionally, a significantly higher percentage of the IG (64.1%) achieved a clinically significant reduction in FP^skf^ compared to the CG (36.2%, p = 0.018). This six-month tailored telephone and email-based exercise intervention induced significant reductions on several measures of body composition in physically inactive adults.

**Trial registration:**

ClinicalTrials.gov (NCT03164239).

## Highlights

• The intervention induced reductions in weight, waist circumference and fat mass
• A total of 64.1% achieved clinically significant reductions in fat mass
• Increasing physical fitness may be important in promoting loss in fat mass.
• This intervention design may potentially reduce the increasing obesity prevalence.

## 1 Introduction

Worldwide, the prevalence of obesity has increased drastically the past few decades and has become a major public health challenge (Ng et al., 2014). Ortega et al. (2017) emphasize the necessity of implementing lifestyle interventions as important measures for reducing the health challenges of obesity. The implementation of modern technology has generated effective modes of enhancing health behavior change (i.e. physical activity, healthy diets and weight reduction) (Allen et al., 2014; Direito et al., 2017; Liu et al., 2015; Tang et al., 2016; Vandelanotte et al., 2016), which are less extensive and less resource demanding compared to more traditional modes (Allen et al., 2014). Additionally, several recently conducted systematic reviews and/or meta-analysis (Allen et al., 2014; Liu et al., 2015; Tang et al., 2016) conclude that technology assisted interventions, may indeed induce significant weight loss, in overweight and obese adults.

Even though previous studies using alternative modes of reach have shown to be promising strategies to reduce weight (Allen et al., 2014; Liu et al., 2015; Tang et al., 2016; Conn et al., 2014), the need for further high quality research in the area is warranted (Allen et al., 2014; Liu et al., 2015; Tang et al., 2016; Vandelanotte et al., 2016; Conn et al., 2014; Mastellos et al., 2014). This is partly due to the difficulty of drawing conclusions based on the wide variation in intervention components, such as study design (Tang et al., 2016), the use of self-reported measures of primary outcome (including self-reported weight loss) (Tang et al., 2016; Mastellos et al., 2014), small sample sizes (Allen et al., 2014; Liu et al., 2015; Tang et al., 2016), inconsistency in the reported effectiveness of different interventions (Mastellos et al., 2014) and partly due to poor study quality (including poor reporting) (Tang et al., 2016; Vandelanotte et al., 2016; Conn et al., 2014; Mastellos et al., 2014). Some of the highlighted limitations concerning previous studies is the lack of information on quality related intervention components (i.e. allocation, blinding, randomization, intervention components, and bias) (Tang et al., 2016; Mastellos et al., 2014). Furthermore, the majority of previous studies report only weight change (kilograms), weight (kilograms), or BMI as primary weight outcomes (Liu et al., 2015; Conn et al., 2014). Weight based indexes may be biased, mostly due to the reduced ability of such indexes to distinguish between fatty tissue and greater-than-average muscularity or skeletal tissue (Sardinha & Teixeira, 2005). Liu et al. (2015) and Allen et al. (2014) clearly emphasize the need to better understand the effect of mobile based weight loss/weight management interventions on waist circumference (WC) and body fat percentage. Additionally, Ortega et al. (2017) emphasize the importance of focusing on cardiorespiratory fitness (CRF), rather than solely on weight/fat loss, as CRF has been found to attenuate the adverse health related consequences of obesity. However, the changes induced by CRF may not provide the same metabolic stress, body compositional changes and thereby the same changes in health status as resistance training does (Clark & Goon, 2015; Clark, 2015). Therefore, rather than focusing on creating energy imbalance, the importance of implementing resistance training in addition to CRF (Ortega et al., 2017) as part of the total exercise program for overfat individuals (Clark & Goon, 2015; Clark, 2015)has been highlighted (Clark, 2015). Thereby, there is a need for adequately reported randomized controlled trials, investigating the effect of exercise as main intervention focus delivered by alternative modes on various objective measures of body composition.

The aim of the present study was to assess the effect of a tailored telephone and email-based exercise intervention on various measures of body composition in a sample of apparently healthy and initially physically inactive adults.

## 2 Methods

The present study was a regional parallel-group randomized controlled trial (RCT), approved by the Regional Committees for Medical and Health Research Ethics (REC) South East D (ref. no. 2010/2371-1) and registered in clinical trials (NCT03164239). Written consent forms were obtained from all participants prior to enrollment and reporting of the trial adheres to the CONSORT statement (Moher et al., 2010) (Appendix A) and TIDieR (Hoffmann et al., 2014). The study was based at, and baseline and posttest outcomes were assessed in a test laboratory, at the University of Agder, Norway.

### 2.1 Sample

Prior to commencing the intervention study in which this paper is based upon, a power analysis was conducted. The main outcome measures were ventilatory threshold (VO_2_max) and fat percentage, though only the results from the fat percentage measures are presented in this paper. The estimated effect size for VO_2_ max was 0,69 mL kg^−1^ min^−1^ (3.3 mL kg^−1^ min^−1^/4,8) (Kelley & Kelley, 2006) and the estimated effect size for fat percentage was −0.36% (−1,4%/3,9) (Kelley & Kelley, 2006), where the measuring instrument for fat mass (InBody 720) showed an intra-class correlation coefficient (ICC) of 0.995 (p = 0.05). Given a power of 90%, and an alpha value of 0.05, a total of 44 participants were estimated to be needed in each group to detect differences of these sizes. Additionally, a previously reported predicted drop-out rate of <20% (Richards et al., 2013), was calculated in to the sample size estimation, rendering a target sample size of 110.

Participants were enrolled between January and March 2011 through four recruitment procedures; 1) participants from the two Agder counties who took part in a national cross sectional study (KAN study) (Edvardsen et al., 2013) were invited by letter to participate (n = 37, 40–55 yr), 2) a random additional sample from the KAN study (Edvardsen et al., 2013), (n = 200, 43–48 yr), were invited by letter to participate, 3) advertisements in local papers and radio, and 4) employees at the University of Agder and Kristiansand community were invited via advertisements on the intranet. The following inclusion criteria were set; the participants had to a) be physically inactive, as in *not* fulfilling the national recommendations of 30 min of daily physical activity set by the Norwegian Directorate of Public Health (2010), assessed by the International Physical Activity Questionnaire- short form (IPAQ-SF) (Craig et al., 2003), b) be within the age range of 40–55 years, c) live in one of the two Agder counties and d) be healthy enough to be able to go through the health related physical fitness assessment. When in doubt whether the participants fulfilled the inclusion criteria of being physically inactive based on IPAQ-SF, the baseline results from the VO_2_max test were compared to normative VO_2_max values published by Shvartz and Reibold (Shvartz & Reibold, 1990). All participants who scored below average by gender and age group, where included in the study.

**Fig. 1:**
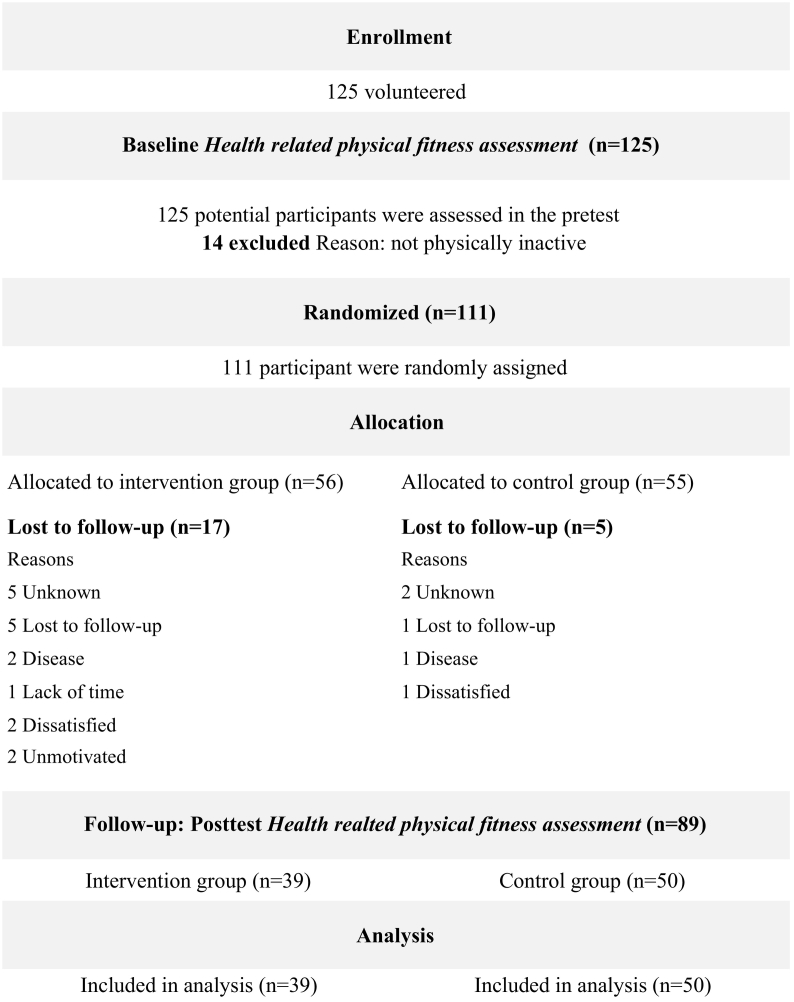
The flow of participants throughout the intervention period (Agder, Norway, 2011).

A total of 125 females (n = 81) and males (n = 44) volunteered to participate in the RCT, where 14 (7 females) were excluded due to them not fulfilling the criteria for being physically inactive. A third party with no further connection to the study randomly allocated all remaining 111 participants to either the intervention group (IG) or the control group (CG), by allocation numbering in SPSS, stratified by gender. A total of 39 participants in the IG and 50 in the CG completed the health-related physical fitness assessments both at baseline and post-intervention (Fig. 1), rendering a total drop-out rate of 19.8%. The project coordinator, data collectors, outcome adjudicators and data analysts were blinded for group allocation. This was ensured through delegating the coordination of practical aspects of the intervention to the counselor, who had no further involvement in the study.

### 2.2 Study design

**Fig. 2:**
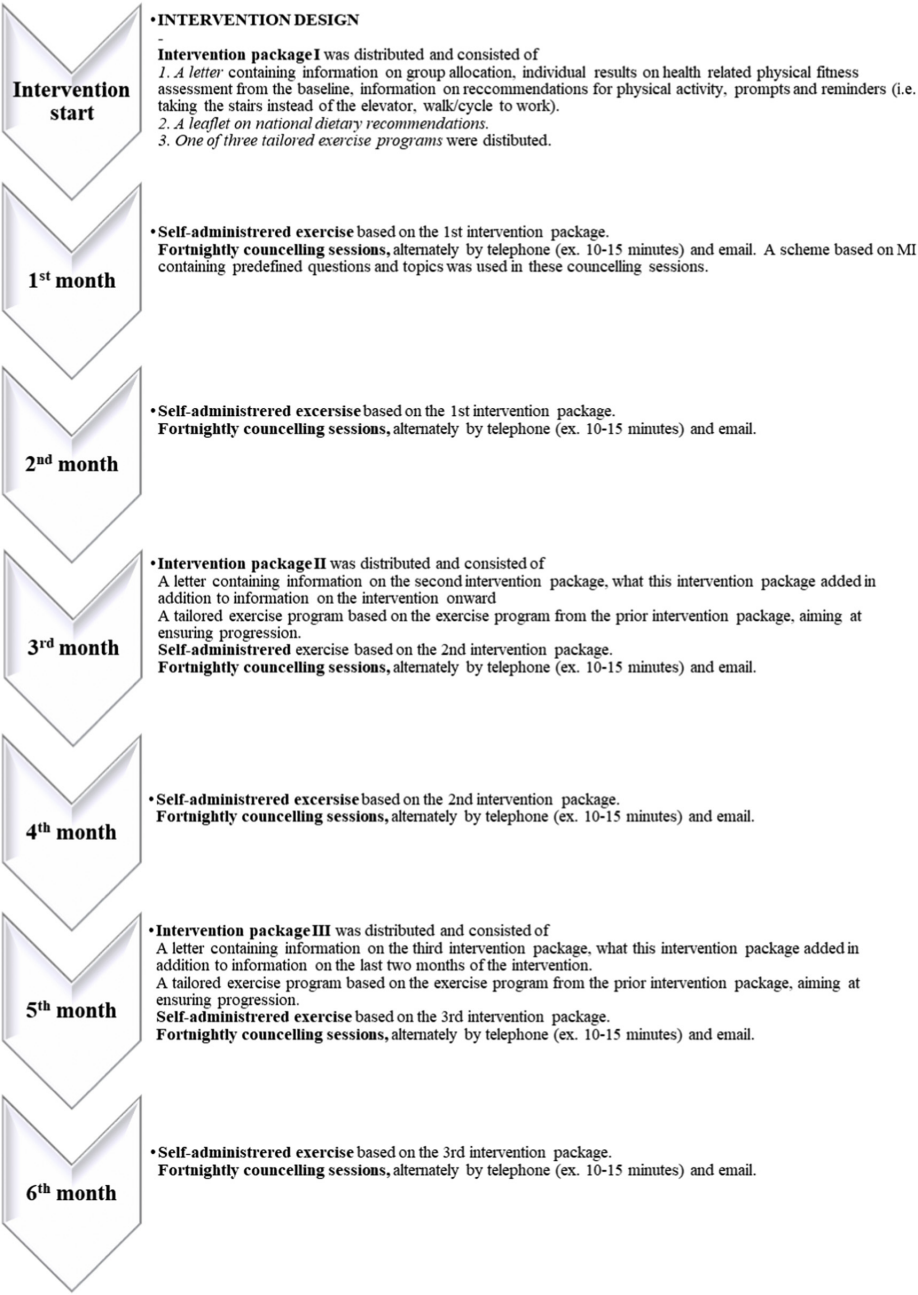
Flow chart of the intervention (design synopsis) (Agder, Norway, 2011).

The intervention duration was set to six months based on financial- and time related aspects. Thereby, the intervention commenced in April 2011 and was ended in October 2011. The intervention received by the IG consisted of: 1) Tailored self-administered exercise recommendations which were developed based on national- and international recommendations for physical activity (Haskell et al., 2007) and a national Norwegian report on physical inactivity (Ommundsen & Aadland, 2009), and 2) Fortnightly counselling sessions, based on motivational interviewing (MI) (Miller & Rollnick, 2002) given alternately by telephone and/or email by a trained person with a master's degree in sport sciences (Fig. 2).

**Fig. 3:**
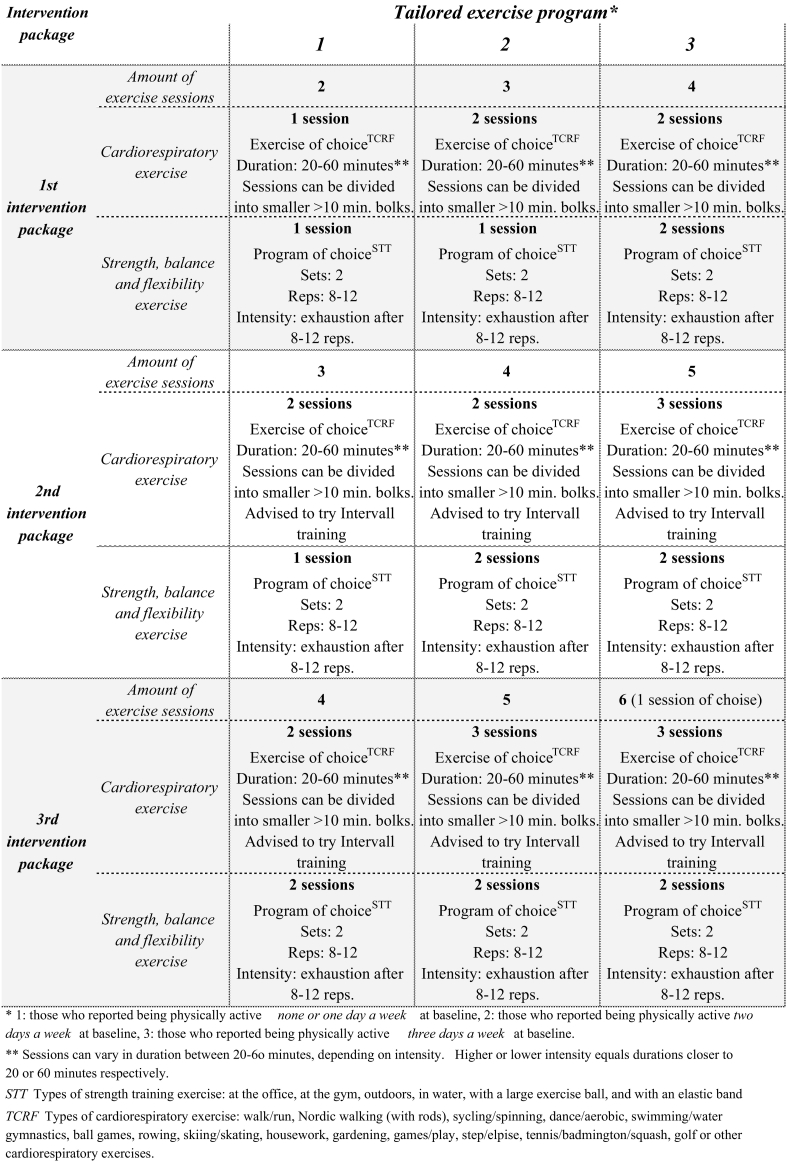
A schematic overview of the tailored exercise programs (Agder, Norway, 2011).

The tailored self-administered intervention consisted of three intervention packages which either were distributed by email or post every two months (Fig. 2), aiming at facilitating the self-administered intervention, ensuring periodization and progress, enhancing motivation and preventing the high drop-out rates previously experienced in some electronic and mobile health interventions (Direito et al., 2017). The first intervention package consisted of written feedback on the participants health related physical fitness (including baseline results on body compositional measures, CRF and musculoskeletal and neuromotor function), a letter containing information on national and international recommendations for- and health benefits of physical activity (Haskell et al., 2007), a leaflet on national dietary recommendations (The Norweigan Directorate of Health, 2011), prompts and reminders in addition to one of three exercise programs (Fig. 3). In addition, the IG was encouraged to increase their physical activity level, associated with daily living. Furthermore, they were given advise on how to acquire exercise equipment if needed (either by buying or by means of utilizing household equipment) and they were informed of possible anticipated variations in physical activity level due to chance or unforeseen events (such as periods of less involvement in physical activity, due to unpredictable reasons, i.e. sickness, lack of time and similar). The three different exercise programs were developed based on how many days per week the participants reported being physically active at baseline, aiming at ensuring tailoring to individual needs. This categorized the participants in one of three predefined physical activity levels: encouraging two, three or four bouts of exercise a week. Based on this categorization, the participants were recommended to increase their physical activity levels with one day from reported baseline physical activity level, except for those who reported not engaging in any form of physical activity, who were encouraged to increase their physical activity level with two days. The second and third intervention packages were distributed two and four months into the intervention period, respectively, and recommended a further one-day increase in physical activity level (Fig. 3), to accompany the progression in physical activity level. The exercise programs included recommendations of both cardiorespiratory-, musculoskeletal- and neuro motor exercises. Each exercise program consisted of various examples of cardiorespiratory exercise and seven different strength training exercise programs for different types and arenas of exercise, developed using EXORLive (EXORLive, 2016). The CG was only informed of the result of the random allocation, and received no follow-up during the intervention period. However, they received similar follow-up as the IG, after the completion of posttest assessment.

### 2.3 Measuring methods

All objective tests were conducted, coordinated and lead by the project coordinator. Baseline and posttest assessments were conducted between mid-March 2011 to mid-April 2011 and between mid-October throughout November 2011, respectively. The main outcome measures for body composition in the present study were: body weight, height, WC and skinfold thickness. Additionally, BMI, body density and fat percentage by skinfolds (FP^Skf^) were calculated. Initially, InBody 720 (Biospace, Korea) was planned used to assess various body compositional measures. However, due to varying results of the instruments ability to give reliable and valid measures of body composition (Volgyi et al., 2008), the results from this test are not reported. All body compositional measures were completed by a trained, ISAK certified investigator, who completed both baseline and posttest assessment, to reduce possible inter-rater bias. Further test personnel were a physiotherapist and qualified master students, whom all had gone through thorough instructions prior to testing in addition to on-set follow-up during testing. Additionally, none of them had any knowledge of or further involvement in group allocation. Measures were conducted using calibrated measuring devices and following a detailed test protocol.

*Body weight* was measured to the nearest 0.1 kg (kg) on InBody 720 (Biospace, Korea), with the participant wearing a t-shirt and underwear. *Height* was measured to the nearest centimeter (cm), using a Seca stadiometer, while the participant was standing without shoes and with the heels touching the wall. *BMI* was calculated using the following formula: body weight/body height^2^ (kg/m^2^).

*WC* was measured by a protocol developed by the WHO (World Health Organization, 2008), where the mid-point between the upper most lateral part of the iliac crest and the lowest most lateral point of the ribcage was set as the marking point for measuring WC values. Two measures were recorded at the end of the participant's expiration using a measuring band and the mean of these two measures were recorded to the nearest half-centimeter.

*Skinfold thickness* was measured using a Lange skinfold caliper (Beta Technology Inc.). The following three sights were measured in males; chest, abdomen and thigh and the following three sites were measured in females: triceps, suprailiac and thigh. All sites were measured two times, and the mean of two measures was recorded to the nearest millimeter (mm) and body density was calculated using the summed skinfold value (Jackson & Pollock, 1978; Jackson et al., 1980; Nevill et al., 2008). The Siri equation (Siri, 1956) was further used to calculate fat percentage based on the body density estimates.

The BMI and WC values were categorized based on WHO developed cut-off values (World Health Organization, 2000; Han et al., 1995). BMI values of <18.5 kg m^2^ were categorized as underweight, 18.5–24.9 kg m^2^ as normal weight, 25.0–29.9 kg m^2^ as overweight and BMI values of ≥30.0 kg m^2^ were categorized as obese. For WC, males and females were categorized as abdominally overweight when WC values were ≥94 cm and ≥80 cm, respectively, and as abdominally obese when WC values were ≥102 cm and ≥88 cm, respectively. As only one participant was categorized as underweight based on the BMI measures both pre (BMI = 18.3 kg/m^2^) and post intervention (BMI = 17.8 kg/m^2^), this participant was included in the normal weight group in all analyzes.

Cut-off values developed by Lohman et al. (1997) were used in order to define obesity based on FP^Skf^, where females and males with FP^Skf^ values >35% and ˃25% (35–55 yr age group), respectively, were defined as obese.

Age, gender and educational level were assessed through a questionnaire as secondary outcome measures. Four categories of educational level were created based on an eight-answering option question: completed less than High School, completed High School, completed three years or less at College/University and completed four years or more at College/University. Physical activity level was assessed by The International Physical Activity Questionnaire short form (IPAQ-SF), where metabolic equivalent of task values inn minutes (MET minutes) were calculated (IPAQ Research Committee, 2005). A difference value was created for the physical activity data, where baseline measures were subtracted from the posttest measures in order to create a delta value for physical activity. A total of 50 (IG: 24, CG: 26) of 89 cases were found valid for analysis by the IPAQ-SF. Additionally, adherence to the counselling sessions was recorded by the counselor.

### 2.4 Statistical analysis

The main analysis was a per protocol analysis, applying a one-way ANCOVA, adjusted for baseline scores and adjusted for gender, age, and educational level. To detect between group differences in posttest values. In order to check for between group differences in baseline data, an independent samples *t*-test was applied for continuous variables, a chi^2^ test for categorical variables, and for continuous and non-normally distributed variables, a Man Whitney *U* test was run. A dependent samples t-test was run to investigate within group differences from baseline to posttest measures for both groups separately, and a Mcnemar analysis was run in order to detect within group changes for the categorical variables and non-normally distributed data. In order to check for between group differences in how many participants achieved a 5% clinical significant reduction on all body compositional measures (Williamson et al., 2015), a chi^2^ test was applied. Additionally, to adjust for gender, age, educational level and baseline fat percentage, a logistic regression was applied. The data was processed using the Statistical Program for Social Sciences (SPSS Version 24.0. Armonk, NY: IBM Corp.). The significance level was set to 0.05.

## 3 Results

A total of 89 participants completed both baseline and posttest and all participants in the IG adhered to the counselling sessions. All participants were either overweight or obese on one or more of the following body compositional measures: BMI, WC, and/or FP^skf^ at pretest. The prevalence of overweight and/or obesity based on BMI, WC, and FP^skf^, ranged from 76.9% (BMI) to 87.2% (WC) in the IG and from 74.0% (WC) to 77.6% (FP^skf^) in the CG (Table 1). The participants in the IG were significantly more physically active at baseline compared to the participants in the CG (p = 0.014). The participants who dropped out from the CG were significantly less physically active at baseline (median: 0 MET min.), compared to the participants in the CG who completed the study (median: 358 MET min., p = 0.017). Furthermore, the participants in the IG who dropped-out of the IG were 3.4 years younger than the completers in the IG (p = 0.01).

**Table 1:** Sample characteristics, by intervention group and control group (Agder, Norway, 2011).

	Intervention group	Control group
	n = 39	n = 50
Age (years), mean (SD)	48.4 (4.6)	47.3 (3.9)
Gender, female%	66.7%	68.0%
Education level		
<high school	2.6%	6.3%
High school	35.9%	35.4%
College/university < 4 yr	17.9%	29.2%
College/university ≥ 4 yr	43.9%	29.2%
Physical activity level (MET min.), median(Q1–Q3)	505.5 (240.0–933.0)⁎	358.0 (66.0–628.5)⁎
Weight (kg), mean (SD)	87.5 (18.9)	88.2 (20.7)
BMI (kg/m^2^), mean (SD)	28.9 (4.4)	29.0 (5.9)
Overweight	35.9%	30.0%
Obese	41.0%	46.0%
WC (cm), mean (SD)	96.3 (14.2)	96.0 (15.2)
Abdominally overweight	28.2%	14.0%
Abdominally obese	59.0%	60.0%
FP^skf^ % obese	87.2%	77.6%

⁎ p for between-group differences < 0.005.

Both the IG and the CG increased their physical activity level significantly from baseline to posttest. The median change in physical activity level for the IG (1239.5 METmin) was significantly higher, compared to that achieved by the CG (194.3 METmin) (p = 0.014). All mean body compositional test scores for the IG decreased significantly from pre- to post test (p < 0.001) (Table 2). The CG decreased significantly in mean scores on WC and FP^skf^ (p ≤ 0.013) (Table 2). When adjusted for pretest scores, the IG decreased significantly more on weight, WC and FP^skf^, compared to the CG (p ≤ 0.046, Table 2), with effect sizes ranging from small to medium (Cohens'd = 0.044) on both the change in weight (p = 0.046) and the change in WC (p = 0.045), to medium (Cohens'd = 0.50) on the change in FP^skf^ (p = 0.010).

**Table 2:** Mean test scores (±standard deviation) for the intervention group and the control group on all body compositional test scores, both at baseline and posttest (Agder, Norway, 2011).

	Intervention group			Δ %	Within group differences (p-value)	Control group			Δ %	Within group differences (p-value)	Between group differences⁎ (p-value)	Effect size (partial η^2^)	Cohen's d	Between group differences (p-value)⁎⁎	Effect size (partial η^2^)
	n	Pretest	Posttest			n	Pretest	Posttest							
		Mean (±SD)	Mean (±SD)				Mean (±SD)	Mean (±SD)							
Weight (kg)	39	87.5 (18.9)	84.7 (17.8)	−3.3%	<0.001	47	86.5 (19.9)	85.5 (20.3)	−0.1%	0.128	0.046	0.047	0.44	0.040	0.053
BMI (kg/m^2^)	39	28.9 (4.4)	28.0 (4.2)	−3.1%	<0.001	42	29.0 (5.6)	28.6 (5.5)	−1.4%	0.086	0.120	0.031	0.36	0.111	0.034
WC (cm)	39	96.3 (14.2)	92.7 (13.3)	−2.7%	<0.001	48	95.2 (15.1)	93.6 (14.8)	−1.8%	0.013	0.045	0.047	0.44	0.037	0.054
FP^skf^ (%)	39	39.1 (7.9)	35.7 (8.8)	−8.7%	<0.001	47	38.7 (9.9)	36.9 (10.3)	−4.7%	<0.001	0.025	0.059	0.50	0.033	0.057

η^2^ eta squared.

⁎ Differences between groups (IG/CG) in post test scores, adjusted for pretest scores.

⁎⁎ Fully adjusted model, adjusted by age, gender and educational level.

The percentage of participants achieving a reduction in body compositional measures during the intervention period varied from 71.8% (BMI) to 87.2% (FP^skf^) in the IG and from 59.6% (BMI) to 74.5% (FP^skf^) in the CG. The IG displayed a significantly higher percentage of participants achieving a clinically significant reduction in FP^skf^ of ≥5%, compared to the CG (Table 3). The adjacent odds ratios for the between group difference in the percentage of participants who achieved a ≥5%reduction in FP^skf^, when adjusting for baseline FP^skf^ and when adjusting for baseline FP^skf^, gender, age and educational level was 3.60 (p = 0.007) and 3.62 (p = 0.011) respectively.

**Table 3:** Displaying the prevalence of participants achieving a clinically significant reduction in body compositional measures of ≥5% (Agder, Norway, 2011).

	Intervention group	Control group	p-Value
Weight	30.8%	12.8%	0.076
BMI	28.2%	16.7%	0.327
WC	28.2%	12.5%	0.117
FP^skf^	64.1%	36.2%	0.018

## 4 Discussion

The main findings from the present study revealed that a six-month tailored email- and telephone-based exercise intervention induced significantly larger reductions on all body compositional measures in the IG compared to the CG, when adjusting for pretest scores in addition to gender, age, and educational level, except for BMI, when adjusting for pretest scores only. The associated Cohens'd (Cohen, 1988) effect sizes ranged from between small and medium to medium.

No previous studies were found having conducted the same type of intervention. However, results from previous recent reviews of self-directed interventions to promote weight loss (Tang et al., 2016), motivational physical activity interventions to promote reductions in body compositional measures (Conn et al., 2014) and mobile phone interventions to promote weight loss in overweight and obese individuals (Liu et al., 2015) reveals that such interventions are capable of promoting significant weight loss (Liu et al., 2015; Tang et al., 2016; Conn et al., 2014), in addition to reductions in BMI (Liu et al., 2015; Conn et al., 2014) and WC measures (Tang et al., 2016). The reductions in body compositional measures and the associated effect sizes and/or standardized mean differences reported by Tang et al. (2016), Conn et al. (2014) and Liu et al. (2015)) were slightly lower compared to the reported reductions in the present study, except for the weight reduction reported by Tang et al. (2016), which was similar. The comparison of our results to that of previous studies with similar design may reflect a superior effect of the present study's design, however, this is uncertain. There is a lack of high quality interventions (Tang et al., 2016; Vandelanotte et al., 2016; Conn et al., 2014; Mastellos et al., 2014) and the variation in delivery mode and study design is large (Allen et al., 2014; Tang et al., 2016; Conn et al., 2014), so comparisons are therefore difficult to make, and possible conclusions are even more difficult to draw. However, the significantly higher prevalence of individuals in the IG achieving a clinical significant reduction (≥5%) in fat percentage (64.1%), compared to the CG (36.2%), supports the effectiveness of the presented study design.

As previously reported (Tang et al., 2016), BMI was the only body compositional measure that was not significantly different between our two groups, when adjusted for baseline BMI measures, only. This may be explained by the nature of the measure, and its reduced ability to distinguish between fatty tissue and greater-than-average muscularity or skeletal tissue (Sardinha & Teixeira, 2005). Thereby, improvements related to increased resistance training and the subsequent relation to health may not be reflected in changes in BMI classifications (Clark & Goon, 2015). Hence, the importance of applying objective measures of fat related body mass beyond that of weight and BMI, in order to enhance better quality in reporting, should be stressed.

### 4.1 Study limitations and strengths

The main strength of the present study is the inclusion of sought for (Allen et al., 2014; Liu et al., 2015; Tang et al., 2016; Mastellos et al., 2014) objective and more diverse measures for assessing body composition, including both WC and FP^skf^ in addition to the more commonly used weight and BMI measures. Although the anthropometric measures used may have measurement errors which must be considered when interpreting the results, all anthropometric measures were conducted by the same trained investigator, at both baseline and posttest.

Secondly, the current study incorporated behavior change strategies and elements that have previously been found to be effective in similar studies such as: goal-setting (Allen et al., 2014; Tang et al., 2016; Conn et al., 2014; Samdal et al., 2017), self-monitoring (Allen et al., 2014; Conn et al., 2014; Samdal et al., 2017), feedback (Allen et al., 2014; Tang et al., 2016; Samdal et al., 2017), prompts and reminders (Conn et al., 2014; De Leon et al., 2014), focus on maintain routines (making habits) (Morgan et al., 2016), information on perceived improvements (including disappointment of experiencing failure to achieve set goals) (Morgan et al., 2016), linking for gym-based activities (Morgan et al., 2016), range of different types of physical activities (arranging for various exercise in variable settings) (Morgan et al., 2016), progression and periodization (Clark & Goon, 2015; Clark, 2016), the use of the transtheoretical model (TTM) (Conn et al., 2014), individualized and personalized service (including tailoring) (Clark & Goon, 2015; De Leon et al., 2014; Morgan et al., 2016; Clark, 2016), personal support and supervision from providers including coaching (Allen et al., 2014; Tang et al., 2016; Samdal et al., 2017; De Leon et al., 2014; Morgan et al., 2016), including a minimum of five contacts during the intervention period (Vandelanotte et al., 2007), and the use of person centered and autonomy supportive communication methods such as motivational interviewing (Samdal et al., 2017).

Furthermore, our study focused on increasing both musculoskeletal and neuromotor fitness in addition to CRF during the intervention period. A recently published meta-analysis of interventions aiming at enhancing health and body compositional changes (Clark, 2016) revealed that effect sizes were larger for interventions implementing resistance training or the combination of resistance training and CRF, especially early in the intervention period, which in turn creates larger metabolic stress and thereby creates weight loss promotion and better health status in overweight and obese individuals.

The main limitation in the present study was the drop-out rate. The study experienced a total drop-out rate of 19,9% (IG: 30.4%, CG:9.1%) during the intervention period. This may have affected the power of the analysis's ability to detect effects, additionally, it may reflect natural drop-outs, and/or it may reflect possible limitations of the study design. The latter is difficult to further elaborate on, as only 35% reported a reason for dropping out. It is possible that the difference in physical activity level between those in the CG who completed the study and those who dropped-out, may be of importance. However, the data on physical activity level were based on self-report and only 50 (IG:24, CG:26) of 89 cases were found valid for analysis by the IPAQ-SF, and thereby the data may be biased. However, based on the reasons given for dropping out, and feedback from the counselor conducting the counselling sessions, the excess work associated with self-monitoring registration of accumulated exercise, and goals prior to the counselling sessions, may have been perceived as too demanding.

Secondly, the duration of the intervention was six months which exceeds the minimum duration recommended for an intervention (Groeneveld et al., 2008). Williams et al. (2015) further emphasize that lifestyle intervention durations of more than six months may not be necessary as weight loss seems to plateau around this period in time. However, a follow-up period should be conducted, to investigate the long term effects of the reported effective intervention (Liu et al., 2015; Tang et al., 2016; De Leon et al., 2014). Adherence to the counselling sessions was registered, but there was no objective registration of adherence to the recommended exercise prescribed. Hence the degree to which the participants adhered to the study design is not known. Furthermore, baseline and posttest assessment were conducted within <2–3 weeks prior to- and after intervention commencement and intervention end, respectively. Additionally, type of exercise in which the participants adopted was not tracked.

In conclusion, the studied intervention design showed promising results, displaying significantly larger reductions in weight, WC and FP^skf^ for participants following the intervention in addition to a larger prevalence of participants achieving clinically significant reductions in FP^skf^ in the IG, compared to the CG. This study design contains various elements which are regarded as success criteria, both based on the results from the present study and based on previous findings. Thereby, this type of study design should be further investigated as to what elements are more effective than others, whether or not elements could be implemented to enhance effect and adherence, and to assess maintenance effects in terms of long term follow-up assessment (De Leon et al., 2014). Additionally, implementing interventions that have shown to have positive effects in primary care settings is of major importance, as this may have large beneficiary consequences for underlying comorbidities associated with overweight and obesity (Kushner & Ryan, 2014).

The following are the supplementary data related to this article.

Appendix A CONSORT 2010 checklist of information to include when reporting a randomised trial*. Appendix A

## Funding

This research did not receive any specific grant from funding agencies in the public, commercial, or not-for-profit sectors.

## Data

Study materials, including materials used in the intervention (intervention packages) can be made accessible by contact with the corresponding author.
